# Exosomal transmission between macrophages and cancer cells: new insights to stroma-mediated drug resistance

**DOI:** 10.18632/oncotarget.26463

**Published:** 2018-12-18

**Authors:** Ziv Gil

**Affiliations:** Ziv Gil: Department of Otolaryngology Head and Neck Surgery, The Laboratory for Applied Cancer Research, The Head and Neck Center, Haifa, Israel; Technion Integrated Cancer Center, Rappaport Institute of Medicine and Research, Technion, Israel Institute of Technology, Haifa, Israel

**Keywords:** exosomes, pancreas cancer, tumor microenvironment, metabolism, miRNA

Although pancreatic ductal adenocarcinoma (PDA) ranks fourth among cancer-related deaths, the cure rate of the disease hardly changed during the last four decades. This dismal prognosis is attributed to two factors: i. Late detection, at a point at which the disease is already metastatic and ii. Resistance of tumors to systemic therapy. However, despite increasing recognition of chemotherapy resistance in PDA, patient outcomes have only minimally improved. Gemcitabine is the workhorse chemotherapy for PDA and for other cancers. It is a cytidine analog that acts to inhibit cell growth by termination of DNA replication. Gemcitabine is metabolized intracellularly by deoxycytidine kinase (dCK) to the active diphosphate (*dFdCDP*) and triphosphate (*dFdCTP*) nucleosides. These nucleosides lead to inhibition of DNA synthesis by two mechanisms: 1. *dFdCDP* inhibition of ribonucleotide reductase (RR), and 2. *dFdCTP* incorporation into DNA, which inhibits further DNA synthesis and induces inter-nucleosomal DNA fragmentation and programmed cell death [1]. Resistance to gemcitabine develops within weeks from initiation of therapy. Traditionally, resistance to chemotherapy was assumed to occur due to intrinsic phenotypes of the cancer cells [2]. However, more than twenty years ago, Fidler and colleagues suggested that the tumor microenvironment may also influence the response to chemotherapy [3]. Twenty years later, Weizman et al. showed that tumor-associated macrophages (TAMs), which are abundant in PDA stroma, can prevent cancer cells from undergoing apoptosis during gemcitabine treatment [4]. This phenomenon is also known as stroma-mediated drug resistance (SMDR). Still, many processes governing SMDR are yet to be defined [5]. Macrophages, which are a main constituent of the pancreatic tumor, are recruited to the tumor microenvironment in response to colony-stimulated factor-1 (CSF-1), which is secreted by invading cancer cells. Pancreatic tumors grown in mice-deficient of C-C chemokine receptor type 2 (CCR2 or CD192), which have reduced macrophage recruitment, are more sensitive to gemcitabine [6]. Macrophages transmit signals that can induce drug resistance by two primary mechanisms: 1. Secretion of soluble factor-mediated drug resistance, and 2. Cell-cell adhesion-mediated drug resistance. The results of Weizman et al. revealed that SMDR does not require cell-cell contact, suggesting the involvement of soluble factors in gemcitabine resistance [4]. A recent paper by Binenbaum et al. revealed the mechanism of communication between TAMs and PDA cells that gives rise to gemcitabine resistance [7]. The authors found that macrophages secrete nano-vesicles that carry molecular signals, which are exploited by cancer cells to support malignant growth. Exosomes are membrane vesicles with diameters of ∼80 nm that originate in the late endosome. Their budding from inward invaginations of endosomal membranes generates intracellular multivesicular bodies (MVBs) [8]. The size distribution of exosomes distinguishes them from larger vesicles that have different biophysical characteristics. Pools of exosomes are packed in the MVBs and are released into the extracellular environment after the fusion of MVBs with the plasma membrane. These vesicles can shuttle proteins, mRNAs and DNA molecules to neighboring cells, and thus serve as mediators of intercellular communication [8-10]. Binenbaum et al. used the LSL-Kras^G12D/+^; LSL-p53^R172H/+^; Pdx-1-Cre (*KPC*) transgenic mouse model of pancreatic cancer, which recapitulates aspects of the human disease, including stromal response and gemcitabine resistance; and a double knockout mouse (Rab27b^-/-^;Rab27a^-/-^), with macrophages that are deficient of exosomal secretion [11, 12]. Tumors grown in Rab27ab-deficient mice had better response to gemcitabine than tumors grown in wildtype controls. Binenbaum et al. showed that most exosomes secreted by TAMs are internalized to the cancer cell cytosol and nucleus. The authors transfected a dsDNA “barcode” to macrophages, and after injection of the macrophages to PDA bearing mice, the barcode was recovered only in cancer cells located in primary tumors and in distant metastases.

Another important finding of this work is that exosomes secreted from TAMs carry a high content of microRNAs (miRNAs), among them miR-365, which was upregulated in cancer cells after uptake of exosomes. Transfer of miR-365, by exosomes to PDA cells, inhibited the effect of gemcitabine; whereas transfer of antagomiR-365 restored the sensitivity to gemcitabine.

Mass spectroscopy analysis revealed that exosomal delivery of miR-365 upregulated pyrimidine metabolism and increased tri-phosphate-nucleotide levels in cancer cells. In turn, increased levels of tri-phosphate-nucleotide upregulated cytidine deaminase (CDA), a key enzyme responsible for maintaining the cellular pyrimidine pool, which also deactivates gemcitabine. Increased tri-phosphate-nucleotide levels also competed directly with gemcitabine for incorporation into the DNA chain, further potentiating resistance.

**Figure 1:**
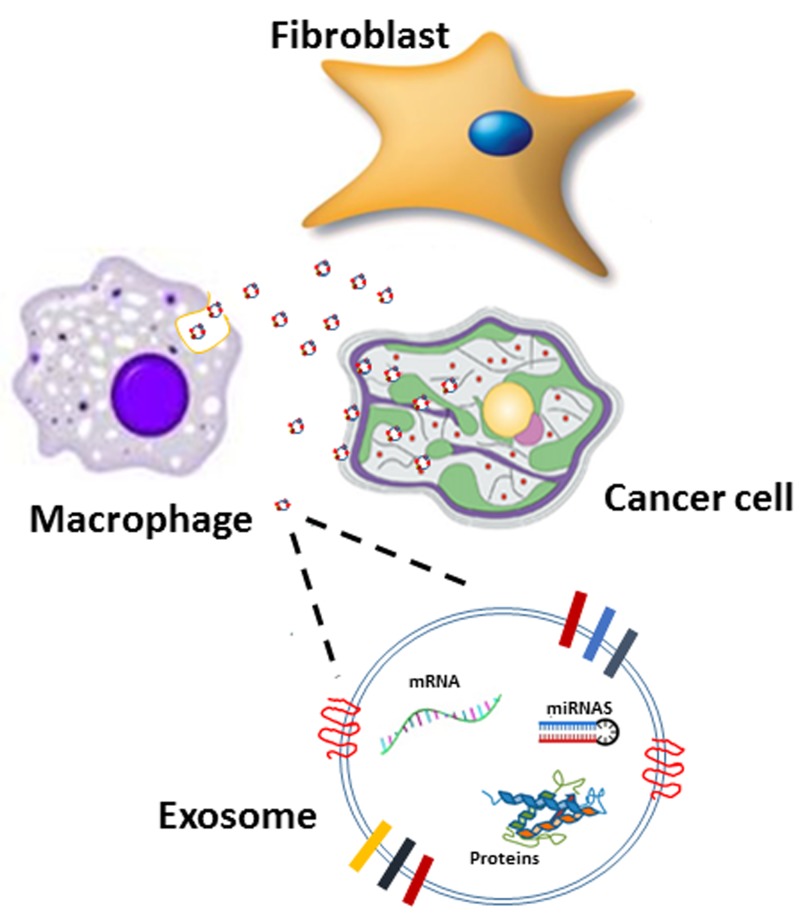
A proposed mechanism for exchange of genetic signals between macrophages and cancer cells Resistance to chemotherapy is induced by macrophages that transfer exosomes to pancreas cancer cells. The exosomal cargo is loaded with miRNA, mRNA and proteins that can modulate the metabolic landscape of cancer cells. Influx of exosomal cargo has the potential to modulate the metabolic landscape of the cancer cell, reducing sensitivity to chemotherapy.

The mechanism by which miR-365 affects the metabolic regulation of cancer cells remains to be elucidated, but previous findings suggested that miR-365 is an oncomiR [13] that acts by targeting nuclear factor I/B (NFIB) [14]. Figure 1 summarizes the proposed mechanism by which macrophages transfer exosomes loaded with miR-365 to PDAC cells, and modulate gemcitabine metabolism.

One implication of the study by Binenbaum et al. is a promising strategy to overcome drug resistance by the immune transfer of antagomiRs to primary tumors via exosomes. This approach resulted in significant improvement in the effect of gemcitabine on the survival of tumor-bearing mice. The knowledge gained from this study is anticipated to be applicable to other cancers for which nucleoside analogues are the treatment of choice.
